# Selections

**Published:** 1892-04

**Authors:** 


### *SELECTIONS.*

---

EXCISION OF THE SYPHILITIC CHANCRE.—Pollitzer, in the New York *Medical Record*, replies to Taylor's article condemning this procedure. (See December issue of this JOURNAL.) Taylor's article was in reply to Jullien's article abstracted in the July issue. Pollitzer's opinion is represented in the report which he submitted as secretary to the section of Dermatology and Syphilis of the Berlin Congress.

It says: "As to the results of the excision of the chancre, there was, with the exception of Neumann, who finds excision useless, complete unanimity. All are agreed as to the theoretical possibility of absorbing syphilis by the early excision of its initial lesion. It gives the patient at least a chance for a mild syphilis which it is incumbent on the physician to offer him." He admits that the histological studies of Taylor and Van Giesen teach that in most cases the excision of a syphilitic growth can only be partial, but also holds that the observations of Auspitz, Unna, Pick and many others go to show that it is perhaps sometimes possible to remove the primary syphiloma entirely. It is also claimed that by excision a certain amount of the infecting material is removed and thus the case made more mild, and the cases of Ehlers and Jullien are quoted to support this. He concludes that there is some probability that a syphilis may be absorbed by an early excision of its initial lesion and a considerable probability that its course will be rendered more benign. Those who have practiced the procedure are asked to publish an accurate account of their cases that a definite opinion may be arrived at as to its value.

Fournier (*L' Union Medicale*) also discusses the abortive treatment of syphilis. Attention is particularly directed to excision. It is an old procedure revived by Auspitz in 1877. He reported thirty-three cases with some successes. Cruvelli, in 1887, collected four hundred and fifty-four observations, and the number not published has reached nearly six hundred.

In excising a chancre an incision should be made a centimetre's distance from it, and the part lifted up on a needle and dissected out. Cocaine has been used, but a general anæsthetic is better. The failures of following this procedure are more numerous than the successes. One finds 162 successes with 309 failures in the statistics of Cruvelli, and 137 successes to 447 failures in more recent statistics, so that one success in five attempts is about the average. This would be quite satisfactory if one were certain of the true character of the cases; but what shall one think of the cases of those who believe in the identity of the soft and hard chancres? Too short a period of incubation also renders the diagnosis doubtful. Some of the cases have been subjected to mercurial treatment, and still others have not been kept under observation long enough. Ten cases of Thierry, with one success, and fifteen of Jullien, with three possible successes, are then adversely criticized. In five cases in which confrontation was possible, and thus the diagnosis placed beyond a doubt, all failed. Mauriac excised a chancre within forty-eight or fifty-six hours without preventing syphilis. Rasori reported a case in which a sore was watched for, and when it appeared was excised within a few hours, but secondaries appeared nevertheless. In concluding, he says that the efficacy of excision, whether as a curative or palliative measure, is a matter of doubt. To excise a case which has induration and glandular involvement is perfectly useless, and has never given a single success. On the contrary, if the chancre has just appeared, one can and should operate so as not to deprive the patient of the least chance of escaping the disease.—*Univ. Med. Mag.*

---

ACUTE URETHRITIS.—Dr. J. W. S. Gouley (*New York Medical Journal*) thus sums up an article on the treatment of acute urethritis :

The study of the nature of urethritis and of the many modes of treatment proposed for its cure has led to the following con clusions:

1. There is no specific for urethritis, notwithstanding the popular belief in its existence.

2. Urethritis cannot rationally be dealt with as a single phlegmasic entity, no matter what may be its cause.

3. The nature, course, and pathic properties of the different stages of the acute types of urethritis indicate that an exclusive method of treatment cannot be carried out in all cases with a reasonable prospect of success.

4. The treatment that is suited to one type or stage of urethritis is often hurtful in another type or stage of the affection.

5. The same therapeutic agent, applicable to a particular type or stage of phlegmasia, is not suitable to all individuals.

6. Balsamics are contraindicated during the first three stages of urethritis, and should not be administered until the fourth or stage of decline is fully established.

7. Urethral injections are contraindicated during the second and third stages of urethritis, but may be used in the first stage and toward the close of the fourth stage.

8. Injections of strong solutions of nitrate of silver, or of strong solutions of any kind, are contraindicated in all the stages of urethritis.

9. Urethritis is ordinarily too much and too vigorously treated. The more heroic and meddlesome the treatment, the greater the liability to accidents and complications, and the longer the duration of the phlegmasia.

10. Confirmed acute contagious urethritis, under the most favorable circumstances and the most judicious treatment, rarely gets well in less than four weeks, except of course in the first attack in young and otherwise healthy men who are overtreated. In the last-named cases it sometimes gets well in ten days or two weeks without medicinal treatment.

11. Proper hygienic management is all-important in the treatment of urethritis; unless it is rigorously carried out, the medicinal and local treatments inevitably fail.

---

COMPARATIVE PATHOLOGY OF THE WHITE MAN AND THE NEGRO.—From an analysis of the cases of 430,466 negro patients treated by the medical department of the American Bureau of Refugees, from 1865 to 1872, Dr. Reyburn, late surgeon United States Volunteers, draws certain conclusions as to the proclivity of the African race to particular types of disease. A basis of comparison between the pathological tendencies of white people and negroes respectively is afforded by 22,053 cases of disease in white patients treated during the same period. Among the negroes there were 152,141 cases of remittent and intermittent fever, and Dr. Reyburn thinks there is no difference as regards susceptibility to these fevers between the white and the colored populations of the Southern States. The statistics further show that the statements commonly made concerning the extreme liability of negroes to scrofulous disease and pulmonary tuberculosis rest on no solid foundation. The deaths from typhoid fever among the negroes amounted to about twenty-five per cent. of the cases treated, this high mortality being dependent on the frequency of severe intestinal lesions. The death-rate from diarrhœa and dysentery was also very high, owing, according to Dr. Reyburn, to the ignorance of hygienic laws which prevails among the colored people. The negro freedman and the white refugee alike fell victims to epidemic cholera, one-half of the patients dying under every variety of treatment. Delerium tremens was of very rare occurrence among the negroes—a circumstance which Dr. Reyburn attributes to "the want of development of the cerebral hemispheres." Alcholism, he says, is in the negro more apt to lead to epileptiform convulsions or mania than to delerium tremens. Dr. Reyburn concludes that the negro has not the same power of resistance to acute inflammations, such as pneumonia, as the Caucasian, and does not recover from protracted and exhausting illness, such as typhoid fever, so well as the latter. On the other hand the negro has greater reparative power after injuries and surgical operations than the white man, in this resembling the other dark races in Asia and elsewhere.—*British Medical Journal.*

---

RELATION OF ALBUMINURIA TO SURGICAL OPERATIONS.—In a paper upon this important theme read before the Southern Surgical and Gynæcological Association (*Virginia Med. Monthly*, Dec., 1891), Dr. Long arrived at the following conclusions:

1. Ether or chloroform rarely injures healthy kidneys.

2. When renal disturbances occur from the use of an anæsthetic, the kidneys being healthy, they are due rather to prolonged narcosis, exposure of the patient, or perhaps to the combined influence of the operation and the anæsthetic.

3. A mild degree of albuminuria (or nephritis), especially if recent, is not a contraindication to the use of chloroform.

4. Even in the presence of advanced and extensive renal changes, an anæsthetic may be employed, provided the patient or the family be advised of the additional risk.

5. Of the two anæsthetics usually employed, it is yet a mooted question as to which is the safer, so far as the kidneys are concerned, unless it be in obstetrical operations.

6. While it is by no means the rule, profound functional disturbance, and even organic lesions may be induced by an operation, apart from the influence of the anæsthetic.

7. Such renal changes are due to reflex sympathetic action, or to sepsis, or both.

8. Operations in certain regions—notably, the abdominal, genito-urinary, anal, or rectal, are especially liable to produce renal complications.

9. A healthy condition of the kidney *minimizes*, but does not obviate, the danger referred to.

10. Albuminuria is always an indication of renal lesions, and should be regarded with distrust, but is not a positive contraindication to an operation.

11. When albuminuria is associated with other evidences of advanced renal changes, no operation should be undertaken without candidly stating to the patient or friends the dangers incident to the condition of the kidneys.

12. Paradoxical as it may seem, an operation will sometimes relieve an albuminuria due to acute affections.

13. No surgeon is justified in undertaking an operation without first knowing the state of the patient's kidneys.—*Med. and Surg. Rep.*

PROPHYLAXIS AND TREATMENT OF INFLUENZA.—Cyrus Edson, of the Health Department of the City of New York, publishes a monograph on *la grippe* and its treatment. Three indications are to be fulfilled: (1) means must be taken to assist the system to rid itself of the poison to which the attack is due; (2) pain must be relieved; and, (3) not the least important, depression must be counteracted. The first indication is obtained by means of castor oil or two compound rhubarb pills. Three or four 3-grain powders of phenacetin are usually sufficient to relieve headache and muscular pains. Salol, 2½ grains to each dose, may be added to the phenacetin with advantage. He deprecates antipyrin and its congeners, which serve to augment the depression, and recommends instead Hoffman's anodyne, which is diaphoretic, diuretic and stimulant. To overcome depression during and after the disease, he recommends the free use of tonics. He repeats Professor Laffont's (of Lille) recommendation of coca preparations, those of Mariani being given the preference. During the disease a hot grog, one-third Mariani wine of coca and two-thirds sweetened water, is administered, taken very hot, several times a day, the slight diaphoresis induced being a valuable addition to the tonic action. (The editor, in the coming issue of the *Annual*, recommends the exhibition of coco in the early stages of the disease, with a view to counteract the impending asthenia and curtail the disease. Six grains of blue mass are first ordered, and, as soon as a couple of movements have been obtained, two tablespoonfuls of Mariani coca-wine are given, every two hours; lozenges, each containing two grains of coca-leaves and 1-12 grain of cocaine, contribute greatly to ward off the pharyngo-laryngeal complications. A 6-per-cent. solution of cocaine, applied occasionally to the nasal mucous membrane, directing the cotton-covered probe toward the roof of the nose and anteriorly, reduces markedly the pain caused by involvement of the frontal sinus. He fully agrees with the author as regards the contraindication of antipyrin.) Edson considers champagne, generous wines, tonic doses of quinine, iron and strychnia also of value. The catarrhal irritation of the air-passages are best allayed by inhalations of compound tincture of benzoin. Chloroform liniment is recommended as a rubefacient; opium and carbonate of ammonia for the cough. The treatment of pneumonic *grippe* is essentially the same as that of uncomplicated pneumonia, the author emphasizing the advisability of preserving the strength of the patients.—*La Grippe and its Treatment.*

---

1. *On the Relative Value of Perineal and Suprapubic Lithotomy.* By Dr. Wilhelm T. Lindenbaum (Jaroslaval, Russia). In the course of the last nine years the author has made seventy perineal lithotomies in children under 15 years of age, with two deaths; and thirty-two in adults, with eight deaths. Besides, during 1890 he performed ten suprapubic lithotomies in patients aged from 8 to 52 years, with one death (the fatal case referred to, a man of 52, with pulmonary tuberculosis and fatty heart). The high operation was conducted after the following rule: 1. All instruments were sterilized. 2. Colpeurynter was introduced into the rectum. 3. The bladder was filled up with 250 cub. cm. of a salicylic solution. 4. Drainage was inserted (no vesical sutures being employed). 5. The patient was kept on his abdomen for from eight to ten first days. 6. The dressing was changed once daily. The urine began to flow through the urethra, on an average, on the twentieth day, the wound soundly healing on the thirtieth. As far as young children are concerned, suturing the bladder is thought to be very difficult, and, on the other hand, quite superfluous, since a healthy urine does not irritate the wound. The author's general corollaries are as follows: 1. Perineal lithotomy in early childhood represents a safe operation and gives excellent results. A relatively enormous percentage of deaths in old age can be explained by the coexistence of grave complications about viscera (especially kidneys). 2. Suprapubic lithotomy does not offer any important advantages over the perineal operation. The mortality remains yet very high, even in children. 3. Still, speaking generally, in the presence of stones, measuring above 2 cm. in diameter, the high section should be preferred, but in cases of smaller calculi perineal lithotomy should be performed.—*Meditz. Obozren.*, *Annals of Surgery.*

PEROXIDE OF HYDROGEN IN DIPHTHERIA.—Dickey (*Ann. Gyn. and Pæd.*) says he knows of nothing in the whole materia medica that will dissolve the diphtheritic membrane so quickly and thoroughly, and yet leave the healthy mucous membrane intact. Dilute it twenty-five per cent. (although it can be used full strength) and apply with an atomizer. This can be repeated until effervescence ceases, when the membrane will be found to have practically disappeared, leaving a whitish surface. If the nose is invaded, it can be applied there with equal satisfaction. Absorb all the watery secretions from the nostrils with blotting-paper or absorbent cotton, and then apply the peroxide. After using the peroxide, use a solution of chloral hydrate, glycerine and water, either as a gargle or with an atomizer. At the same time bichloride of mercury, tincture of the chloride of iron, with or without chlorate of potassium, or such other remedies as may suit the judgment of the individual prescriber, or be applicable to the case in hand, may be used. The writer prefers the bichloride. Coupled with this should be given good digestible food at regular intervals, of which milk should form the basis, and such stimulants, from time to time, as the individual case may demand. The constitutional treatment is not less important than the local, and consequently must be attended to from the outset. When the temperature exceeds 103.5°, sponge the entire body with tepid water as often as may be necessary to bring it below this point. Pellets of ice internally will allay thirst and relieve very materially the turgid condition of the blood vessels, and should not be omitted. Ice may also be applied to the throat in a rubber bag or a bladder, relieving greatly the inflamed glands. —*Popular Science News.*

---

TREATMENT OF SYPHILIS DURING PREGNANCY.—This problem is one which not infrequently presents itself to the practitioner of medicine. A man infects his wife and she becomes pregnant and is menaced with all the dangers incident to conception after such circumstances. Besnier says that the treatment should be energetic, and should consist of tonic and specific remedies.

1. Tonic medication: good food, syrup of iodine of iron, preparation of cinchona.

2. Specific medication: give one of the following pills daily:

| | | | |
|---|---|---|---|
| ℞. | Hydrarg. bichlorid, | . . . | gr. 1-16. |
| | Ext. opii, | . . . . . | gr. 1-12. |
| | Ext. gentian, | . . . . | gr. 1-12. |
| | Glycerini, q. s. | M. ft. tal. . | pil. q. s. |

3. Iodide of potassium should be prescribed at the same time in doses of eight to fifteen grains. This treatment should be continued during the entire period of pregnancy, and the increase in weight of the patient will prove the efficacy of the measure. It may appear to some that the dose of bichloride is not very large, but it must not be forgotton that the treatment is continuous, and moreover women are more susceptible to its action than men.—*St. Louis Medical Journal.*

---

THE CURE OF PHTHISIS.—In a paper on the Processes which Result in the Arrest or Cure of Phthisis in a recent issue of the N. Y. *Medical Record*, Dr. Henry P. Loomis arrives at the following conclusions:

1. Out of 763 persons dying of a non-tubercular disease, 71, or over nine per cent., at some time in their life had phthisis, from which they had recovered.

2. The new fibrous tissue by which the advance of the disease was apparently checked, and the cure effected, developed principally by round-cell infiltration of the interlobular connective tissue, which in some instances had increased to an enormous extent. Some of the new fibrous tissue was formed later by round-cell infiltration in the alveolar walls and around the blood-vessels and bronchi. Pleuritic fibrosis appears to be secondary to tubercular processes in the lung substance. The interlobular connective tissue is the primary and principal source of the fibrosis.

3. Tubercle bacilli were present in the healed areas in three out of twelve of the lungs examined. These healed areas did not differ in their gross or microscopical appearances from those in which they were not found.

4. Thirty-six per cent. of all cases where the lungs were free from disease showed localized or general adhesions of the two surfaces of the pleura.

---

TREATMENT OF SORE THROAT.—It is now generally admitted and agreed that in diphtheria some form of antiseptic or at least aseptic treatment should be used, but for the simpler forms of throat inflammation, and the catarrhal as well as tonsillar troubles, which are supposed to be caused by cold, the treatment remains for certain doctors what it was,—that is, chlorite of potassium or other gargles of an emollient or astringent nature,—and yet the simple fact that these troubles are frequently seen in the same family, and at the same time as diphtheritic diseases, would seem to indicate a common origin. The infectious nature of tonsillitis and other simple forms of throat and bronchial diseases or inflammation seems likely, and once this is suspected, if not proved, it should lead to trying antiseptic methods instead of the old astringents. The following is a formula much used in France:

℞. Acid carbol., cryst.,

Camphor, aa gr. xv;

Glycerini,

Aquæ destill., aa f℥ii.

This is painted on the inflamed part three times a day. It will be found to have a mechanical action as well as an antiseptic one.—*Archives of Pediatrics*, December, 1891.

---

DELIVERY OF THE EXTENDED ARMS IN BREECH LABOR.—The *New York Journal of Gynecology and Obstetrics* mentions the following as the proper manipulations:

1. Twist the child's body so that the shoulder lying nearest the sacrum is carried toward the sacrum.
2. Draw the legs and trunk sharply toward the opposite side and somewhat forward until the scapula is felt. This drags the the elbow down near the brim.
3. Slip in two fingers (or the flat hand) well forward under the pubic arch and reach along the child's humerus to the elbow.

4. Push the arm across the face, and then sweep it down to the chest and across it, and out of the vulva.

5. Rotate the body to bring the remaining shoulder back toward the sacrum, and the liberated arm under the symphysis.

6. Slip the fingers of the other hand under the pubic arch and along the child's arm, and attempt to sweep the elbow past the face.

6a. At the same time the other hand on the supra-pubic region must push the occiput in the opposite direction, so that the head turns on the neck, and elbow and face go over together.

Dr. Dickinson calls particular attention to the last maneuver as one not usually described in the books. The other steps are more or less familiar.

---

TO WHOM IT MAY CONCERN.—For the instruction of some of our subscribers we publish the following laws laid down by the United States Government, for the protection of newspapers and periodicals:

Subscribers who do not give express notice to the contrary are considered as wishing to continue their subscription.

If subscribers order the discontinuance of their periodicals, the publishers may continue to send them until arrearages are paid.

If subscribers move to other places without informing the publishers, and papers are sent to former direction they are held responsible.

The courts have decided that refusing to take periodicals from offices before removing and leaving them uncalled for is *prima facie* evidence of intentional fraud.

If subscribers pay in advance they are bound to notify the publishers at the end of their time, if they do not wish to continue taking it, otherwise the publisher is authorized to send it on and the subscribers will be responsible until an express notice, with payment of all arrears, is sent to the publisher.

Publishers of newspapers can, under law, arrest any man for fraud who takes a newspaper and refuses to pay for it. Under this law it is a dangerous trick for a man to allow his subscription account to run on from six months to a year and then tell the postmaster to mark "refused" or to send the editor a notice to discontinue the paper.

POINTS ON SYPHILIS.—The glands above Poupart's ligament are the immoral glands. If you find them enlarged examine the penis, and in nine-tenths of the cases you will find the cause there. If the swelling is in the glands below Poupart's ligament, the cause is probably in the foot. In syphilitics a heavy chill and high fever, followed by sweating, will be followed by marked secondary symptoms. Secondary symptoms beginning with a papular or tuberculous eruption show a very severe attack. Syphilitic eruptions are polymorphous; that is, many forms of eruptions are present at the same time—the roseolous, erythematous, papular, etc. This is not the case in non-syphilitic eruptions, a point of diagnostic importance. The reason the hair is lost in syphilis is there is a proliferation of connective tissue cells, which press on the hair-bulbs and cut off the blood supply and cause the hair to die. As soon as the patient is put on treatment, and these cells are absorbed, the hair again grows if the bulbs have not been destroyed. It is by means of the skin that the poison of syphilis is eliminated as we see by the eruption.—*Jonathan Hutchinson.*

---

CAMPHOR-MENTHOL IN CATARRHAL DISEASES.—Dr. S. S. Bishop, of Chicago, in the *Kansas City Medical Index*, reports excellent results following the use of a camphor-menthol mixture in nasal catarrh, hay fever, etc. He mixes equal parts by weight of gum camphor and menthol. These together form an oleaginous liquid which he mixes with lanoline in the proportion of twenty of the former to eighty of the latter. Instead of the lanoline liquid albolene may be used. The mixture is thrown into the nostril by means of a good atomizer, and this is followed at once by a blanching of the mucous membrane and a reduction of the swelling and congestion. In violent sneezing, with temporary stenosis of the nostrils, it gives immediate relief. Author reports favorable results also in the treatment of hay fever.

Finally, camphor-menthol contracts the capillary blood vessels of the mucous membrane, reduces swelling, relieves pain and fullness of the head, or stenosis, arrests sneezing, checks excessive discharges and corrects perverted secretions.

THE ETIOLOGY OF CHANCROID.—*The Medical News*, December 5th, contains a valuable paper on this subject by Dr. R. W. Taylor, New York. His conclusions are:

What we call chancroid is the product of many varieties of pus derived from non-syphilitic and syphilitic subjects. It is therefore a hybrid, heterogeneous lesion, in all cases a septic ulcer, and in many instances simply an active form of wound-infection. This septic ulcer in some cases originates *de novo* from the contact of pyogenic microbes with a raw surface, herpetic or eczematous excoriation, a chafe, etc., sexual contact then having nothing to do with its development. As a general rule, this local infective process is more active in syphilitic than in non-syphilitic subjects. It follows, therefore, that so long as pyogenic microbes and tissue-predisposition exist, chancroids will be found upon the mucous membranes and integument of the human race.

---

TREATMENT OF ACUTE BRONCHITIS.—Simple cases usually recover under the use of a good expectorant mixture, such as :

℞. Ammon. muriat., ℥ss.
Mist. glycyrrhiz, Co., ℥iv.

M. Sig.—Dessertspoonful every three or four hours.

When the secretions are abundant, and not easily coughed up a turpentine emulsion is excellent, For instance :

℞. Ol. terebinth, ʒij to ʒiij.

Muc. acaciæ, q. s.

Aq. cinnamon, ʒi.

Aquæ, q. s. ad. ℥vj.

M. Sig.—Tablespoonful in water every four hours.

Sometimes the cough is of such an irritating character that expectorant measures avail little. Some narcotic must then be used. Codeine has not the disadvantages of morphine, and is efficient. A good combination is :

℞. Codeine sulphat., gr. viij.
Syr. prun. virgin., ʒij.

M. Sig.—Tablespoonful in water three or four times per day, and at bed-time if necessary.—*W. B. Canfield, in Therapeutic Gazette.*

GONORRHŒA IN WOMEN.—The more exact studies of the present have expanded very greatly our knowledge of gonorrhœa in women (*Med. and Surg. Rep.*) It is but a little while since this disease in women was considered as trivial; as being only a vulvovaginitis which would recover even without treatment in six weeks; and as meriting attention, not so much because of the evil it might do the women, as because of the possibility of the disease being conveyed to men. Enlarged experience has shown this conception of the affection to be not only inadequate, but even partly erroneous. The error is with reference to the usual seat, which has been shown to be the cervix uteri and not the vagina. It is now well established also, that far from the disease terminating spontaneously it tends to persist in a chronic form for a very long time—almost indefinitely. The disease tends to invade the entire genital tract. Not only the vulvovaginal glands, but the endometrium, tubes, ovaries and the peritoneum—even distant organs and tissues as the knee or ligaments of the spine may be affected. Thus, incurable or even fatal conditions may and frequently do arise from this disease heretofore considered so trivial; not to consider the extension of the disease to the urinary organs, where it may produce the same serious conditions which it does in men.—*Med. Rev.*

---

THE NECESSITY OF STATE MEDICAL EXAMINATIONS.—The salutary effects of State medical examinations are forcibly illustrated in the report of the Medical Examining Board of the State of Washington. Of thirty applications for authority to engage in the practice of medicine, fifteen were rejected. The questions asked were of such a nature that only the incompetent could fail of the requisite average. The report is but a repetition of like reports that have emanated from other States. As a result of restrictive medical legislation, not only is the number of successful applicants reduced, but the actual number of original applicants is also diminished. It is the imperative duty of the State to afford the community adequate protection from the devices and intrigues of quacks and adventurers, with whom reputable men and women cannot compete.—*Medical News*.

THE NECESSITY OF PURE DRINKING-WATER.—It is evident that the necessity of using absolutely pure drinking-water cannot become too strongly impressed on the public mind, but water in that condition is provided by very few communities. Hence the public are availing themselves of bottled natural mineral waters to a great extent. Where such waters cannot be obtained, the ordinary drinking-water, if the least suspicion attaches to it, should be boiled before using. Precautions should be taken at all times of the year. It is often thought that in early spring, when rivers are swollen from melting snow, river water is purer and safer than in summer or fall. Recent experiments, however, have shown that the number of bacteria in the water supply increases greatly while the snows are melting on the uplands. Ice also is known to be a freqeunt source of poisoning; hence, while the water that is used may be pure, the ice that is put into it often renders it noxious.—*N. Y. Med. Journal.*

---

TREATMENT OF EXOPHTHALMIC GOITRE.—M. Fergusson has published (*Bull. Medical*) the good effects obtained by strophanthus in the treatment of exophthalmic goitre. In nine cases in which he employed this drug the almost immediate disappearance of cardiac symptoms permitted the patients to resume their occupations. Pulse fell from 150 and over to between 85 and 75; at the same time there was a diminution in the exophthalmia, and in the thyroid gland to a fibrous condition, the result of the great duration of the affection. M. Fergusson does not affirm that the cure is absolute, but insists that the amelioration is very considerable. The author recommends large doses, 8 to 10 drops of the tincture three times daily. In some cases he has given as much as 20 to 25 drops at a dose. He believes that small doses have no effect whatever.—*Journal de Medecine.*

---

The *Boston Medical and Surgical Journal* states that physicians headed the list of suicides last year, as they have for the past ten.

INTERNAL ANTISEPTICS.—Salol is the best of the internal antiseptics, because it is always well borne by the digestive tract; it is but slightly soluble, and is decomposed into carbolic and salicylic acid only in an alkaline medium, *i. e.*, in the intestine. Iodoform and napthol, which are always toxic and irritant, are much inferior to it, for it is but slightly toxic. Almost equally valuable is the salicylate of bismuth, which acts on both the stomach and intestine.

℞. Salol,
Bismuth salicyl.,
Sod. bicarb., aa ʒ iiss.
Div. in caps. xxx. —*Dujardin Beaumetz, Ex.*

---

CHRONIC OR TUBERCULAR BRONCHITIS.—Dr. James E. Reeves, of Chattanooga, Tenn., has had a very large experience with the following formula, and sends it, in a private letter, with the assurance of its possessing great remedial virtues:

| | | |
|---|---|---|
| ℞. | Chloride of ammonium, . . | ℥i. |
| | Iodide of ammonium, . . | ℥ss. |
| | Glycerine, . . . . | ʒij. |
| | Beechwood creasote, . . | mlxiv. |
| | Fowler's solution, . . | ʒiss. |
| | Phillips' syr. wheat phosphates, q. s. ad., . | ℥viij. |

M. Sig.: A teaspoonful four times a day.—*Columbus Med. Jour.*

---

THE RELATIONSHIP OF SYPHILIS TO ANEURISM.—Is aneurism in comparatively young subjects, say under forty, consequent upon syphilis? Always? No. In very many cases? In all probability. As one of its most marked effects, the specific disease unquestionably produces endarteritis; and, though this inflammation especially attacks the medium-sized and smaller vessels, it is not limited to them, and the weakening of the walls that it produces cannot but predispose to fusiform or sacculated dilatation.—*Dr. P. S. Conner, Medical News, January* 23.

SALICYLIC ACID IN CHANCROIDS.—Dr. Blanc claims that in the treatment of chancroids he has attained better results with salicylic acid than with iodoform, iodole, aristol, calomel, or any of the antiseptic powders usually applied in this affection. His method is as follows: The sores are at first dried with absorbent cotton and then cauterized with pure nitric acid. This produces a superficial slough and destroys the specific nature of the ulcer. There is another advantage in the use of the caustic which should never be lost sight of, namely, that it may also destroy the poison of syphilis if the sore be a recent one; for the syphilitic virus frequently accompanies the chancroidal, and, while there is yet no indication of absorption, there is a possibility that the poison may not have been taken into the system. Salicylic acid will cure many chancroids without the previous use of nitric acid, and its application in this way has been vaunted by Hebra and many others; but, for the reasons given above, it is better to use the caustic. The sore is then dusted with pure salicylic acid, care being taken to put the powder only on the diseased surface. In some cases there is a burning sensation lasting a fraction of a minute, and then there is no more discomfort from the use of the salicylic acid. After four days this is discontinued, and the sore will then heal under any suitable mild powder or salve.

Salicylic acid, besides being a reliable antiseptic, has a direct action on the epithelial cells of the mucous membrane, destroying them and leaving sound tissues beneath. While the powder is upon the sore, the edges of the latter will take on a whitish or blanched appearance, which rapidly disappears when the drug is discontinued.—*The New Orleans Medical and Surgical Journal.*

---

TONSILLITIS.—The *London Medical Recorder* contains the following prescription for the rapid relief of tonsillitis:

| | | |
|---|---|---|
| ℞. | Tinc. of veratrum viride, . . . | mxxx. |
| | Sulphate of morphia, . . . . . | gr.jss. |
| | Distilled water, . . . . . . . . | ʒvj. |
| M. | | |

Sig. One teaspoonful, given twice, with one hour's interval at the outset of the treatment, and then at intervals of two or three hours, as may be required.

LAWSON TAIT ON THE "CORPUS LUTEUM OF PREGNANCY."—Writing to the *Lancet* of January 2, 1892, on this subject, Dr. Tait asserts with characteristic energy:

I am perfectly confident that I have made sections of more ovaries, concerning the actual history of which the facts were known, than any man alive. I have only to say that my conclusion, long ago made and confirmed by every investigation which I have made, is that the belief that the corpus luteum is altered in any way by pregnancy, or that there is such a thing as a true corpus luteum or a false corpus luteum in relation to the fact of pregnancy, is one of the most extraordinary crazes which has crept into medical belief, and it has been productive of a very large amount of mischief. It is one of those assertions which has been made in the early investigation of ovular physiology and pathology, based on the assumption that ovulation is a monthly occurrence, and it has been handed down from writer to writer without any kind of substantiating proof, whilst the negative evidence against it is perfectly overwhelming.—*Kansas Med. Jour.*

---

CHLORAL FOR BOILS.—This treatment ought, according to M. Spohn, to replace all others on account of its efficiency. It consists in covering the furuncle with tampons of antiseptic lint saturated with the following solution :

℞. Chloral hydrate, ʒijss.

Glycerine,

Water, aa ℥x.

—*Journal de Medicine.*

---

INTERTRIGO.—The following ointment is given in one of our exchanges as a good application in chafe.

℞. Acid borici........................gr. vij.

Lanolini........................℥xij.

Vaselini........................ʒiij.

M.

This ointment is to be applied to the diseased area, which is first cleaned by the use of a mild soap.

MEMBERSHIP IN THE AMERICAN MEDICAL ASSOCIATION.—This is obtainable, at any time, by a member of any State or local medical society which is entitled to send delegates to the Association. All that is necessary is for the applicant to write to the treasurer of the Association, Dr. Richard J. Dunglison, Lock Box 1274, Philadelphia, Pa., sending him a certificate or statement that he is in good standing in his own society, signed by the president and secretary of said society, with five dollars for annual dues. Attendance as a delegate at an annual meeting of the Association is not necessary in order to obtain membership. On receipt of the above amount the weekly Journal of the Association will be forwarded regularly.

---

MATERNAL IMPRESSIONS.—Anent the recent birth of a Chinese baby of a white mother in Philadelphia, as a result of maternal impressions induced by a Sunday-school class of Chinese, which the lady was teaching, a good joke is related:

The wife of a physician in this city read the account of the Chinese impression to her husband and asked him his opinion of it. "Humph!" he growled, "possible." "And," his wife resumed, "I read a short time ago, of a lady who had been chased by a negro and was afterward delivered of a negro child. Do you think such a thing could happen?" "Yes," replied our cynical doctor, "if the nigger caught her."—*West. Med. Reporter.*

---

The death of Dr. Standiford, of Chicago, from the effects of alchohol and opium, and the committing to jail for drunkenness of the Secretary of the Bichloride of Gold Club in a Western town, coming so closely after the death of Col. Mines gives rise to the suspicion that the Keeley cure is as fallible as other human inventions, says the *Western Medical Reporter.* Meantime Dr. Keeley's receipts are $3,000.00 a day and the curing goes merrily on, and will probably go on as long as the fool-killer takes a vacation.—*Med. Review.*

